# New York Cattle Quarantine

**Published:** 1883-10

**Authors:** 


THE NEW YORK CATTLE QUARANTINE.—The United States Government has bought forty acres of land at Garfield, New Jersey, having in view the establishment of a quarantine station to which all foreign cattle arriving in the port of New are taken.

The *Country Gentleman* gives the following particulars:

If the animals are found suffering from any fatal contagious disease, as lung plague, rinderpest, or aphthous fever, they must not be admitted to the regular quarantine, but quarantined elsewhere. The cattle seemingly free from all infectious diseases are taken to the Garfield grounds with great care, every precaution being taken to avoid any risks of spreading a disease. Only cattle arriving by the same ship may be quarantined in the same yard, and the rules require that all gates of yards shall be kept locked when cattle are entering or leaving the quarantine grounds. A veterinary inspector has general supervision and reports to the Treasury Cattle Commission the appearance of any contagious disease. As a further precaution the manure is quarantined for three months after the cattle have served their ninety days and then removed from the grounds."

The government has devised, and no doubt will put into execution, a plan for the prevention of the spread of contagious diseases among cattle, the importance of which no one will deny.

We would suggest in the matter that not only the infected animals, but also the men who have been in contact with them be subjected to quarantine regulations.

